# Prediction of Aortic Stenosis Progression Using Artificial Intelligence: A Machine Learning Model

**DOI:** 10.1016/j.jacadv.2025.102121

**Published:** 2025-08-29

**Authors:** Edward Itelman, Yaron Shapira, Alon Shechter, Nadav Loebl, Yuval Altman, Leor Perl, Ran Kornowski

**Affiliations:** a Department of Cardiology, Rabin Medical Center, Petah Tikva, Israel; b Tel Aviv School of Medicine, Tel Aviv University, Tel Aviv, Israel; c Beilinson Medical Center Innovation, Artificial Intelligence Center, Rabin Medical Center, Petah Tikva, Israel; d Faculty of Computer Science, Reichman University, Herzliya, Israel

**Keywords:** aortic stenosis, artificial intelligence, echocardiography, machine learning, risk prediction, valve disease progression

## Abstract

**Background:**

Current guidelines for monitoring aortic stenosis (AS) progression focus on serial echocardiographic assessment, which is resource-intensive and subject to variability. Artificial intelligence may offer an opportunity to enhance the early identification of patients at risk of developing severe AS.

**Objectives:**

The objective of this study was to create an echo-based model that can predict whether a patient will deteriorate from mild/moderate AS to severe AS.

**Methods:**

We retrospectively analyzed a single-center database of 529,751 echo exams and identified 9,330 echocardiograms of patients initially diagnosed with mild or moderate AS, 56% of which progressed to severe AS within 5 years. We developed a model agnostic to any patient data outside the scope of the echocardiography report, and the reports were obtained from a large database of a tertiary medical center. Performance was assessed for accuracy, area under the curve–receiver operating characteristic, and calibration SHapley Additive exPlanations values provided interpretability for the model's predictions.

**Results:**

During the follow-up, 1,625 (47%) patients developed severe AS. The model demonstrated strong predictive performance—an area under the curve–receiver operating characteristic of 0.91, an accuracy of 83%, and an Integrated Calibration Index = 0.0576. The model successfully identified patients at high risk of progression, with robust calibration and generalizability confirmed through cross-validation.

**Conclusions:**

Our novel, echocardiography-focused artificial intelligence model is a reliable tool for the early identification of patients at risk of progression to severe AS. Pending future, multicenter, prospective validation, such models may facilitate personalized follow-up strategies and timely interventions, ultimately leading to improved patient outcomes and resource utilization.

Aortic stenosis (AS) is the most prevalent valvular heart disease in the Western world, with its burden steadily increasing due to population aging. In adults aged ≥75 years, the prevalence of AS is approximately 12.4%, with severe AS affecting about 3.4% of this population segment.^1^ Projections suggest that by 2050, the number of individuals ≥75 years old in Europe will exceed 75 million, with an estimated 9.3 million individuals affected by AS and 2.6 million by severe AS.^2^

Despite the availability of effective therapies, including surgical and transcatheter aortic valve replacement (TAVR), underdiagnosis and undertreatment remain widespread.

Recognizing patients at risk of rapid AS progression is therefore critical. Nevertheless, current guideline-directed follow-up intervals may fail to accommodate individual variability in disease trajectory. According to the 2020 American College of Cardiology/American Heart Association guidelines,^3^ patients with mild AS are recommended to undergo follow-up echocardiography every 3 to 5 years, and those with moderate AS every 1 to 2 years. However, these fixed intervals may be too short or long, as progression rates vary considerably among individuals. Venema et al^4^ showed that faster decline in the aortic valve area (AVA) was independently associated with increased mortality, underscoring the need for tools that personalize follow-up and identify high-risk patients earlier in the disease course. A recent review showed that 1 in 4 patients with moderate or severe AS is not clinically recognized, and rates of missed diagnoses worsen among underrepresented minority groups and women. In population studies, up to 40% of elderly patients with symptomatic severe AS do not undergo any valve intervention.^1^ Généreux et al^5^ reported that only 60.7% of patients with echocardiographically confirmed severe AS underwent TAVR within 4 years, while untreated patients experienced a 4-year all-cause mortality of 44.9%. Similarly, Benfari et al^6^ demonstrated that despite increasing TAVR use over 2 decades, more than 40% of patients with severe AS remained untreated, and mortality rates remained high, with a 3-year rate of 36%.

Delays in diagnosis and, therefore, treatment not only increase mortality risk but also impose significant financial burdens on healthcare systems. In an extensive analysis of Medicare Advantage beneficiaries, patients with deferred TAVR accrued an additional $10,080 in healthcare costs in the first year and over $20,000 within 2 years compared to those receiving timely intervention.^7^ These excess costs were predominantly non-TAVR-related, suggesting worsening clinical status due to delayed care. Similarly, Weintraub highlighted the economic consequences of deferring TAVR, reinforcing that delays may lead to increased resource utilization despite the cost-effective procedure.^8^

The recent advancements in artificial intelligence (AI) and machine learning have paved the way for the early identification and prediction of disease progression in cardiovascular conditions.^9^ AI models have shown great promise in streamlining the analysis of echocardiographic images,^10^^,^^11^ leading to improved diagnostic precision and enabling earlier intervention. This paper details the development and validation process of an innovative AI model designed to predict the deterioration from mild or moderate to severe AS using exclusively echocardiographic data. By harnessing this model, we aim to equip healthcare professionals with a reliable tool to pinpoint patients at risk of rapid progression, facilitating timely clinical decision-making and triage to appropriate treatment and potentially impacting the current disparities in diagnosis rates among women and minority groups (Central Illustration).

**Central Illustration:**
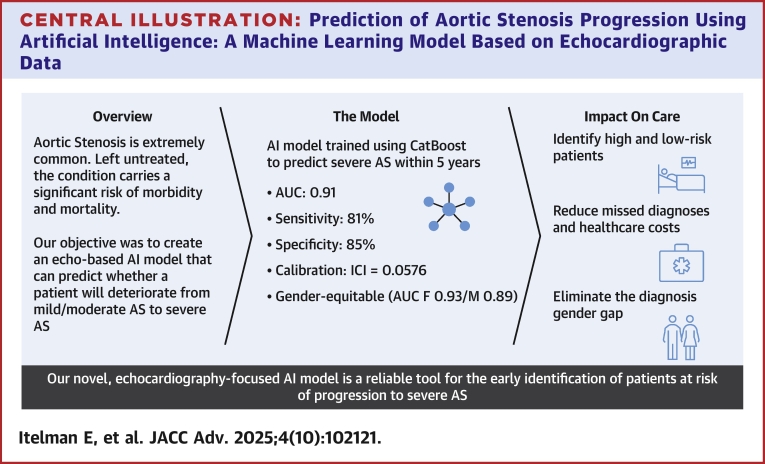
Prediction of Aortic Stenosis Progression Using Artificial Intelligence: A Machine Learning Model Based on Echocardiographic Data

## Methods

### Study population

**Figure 1:**
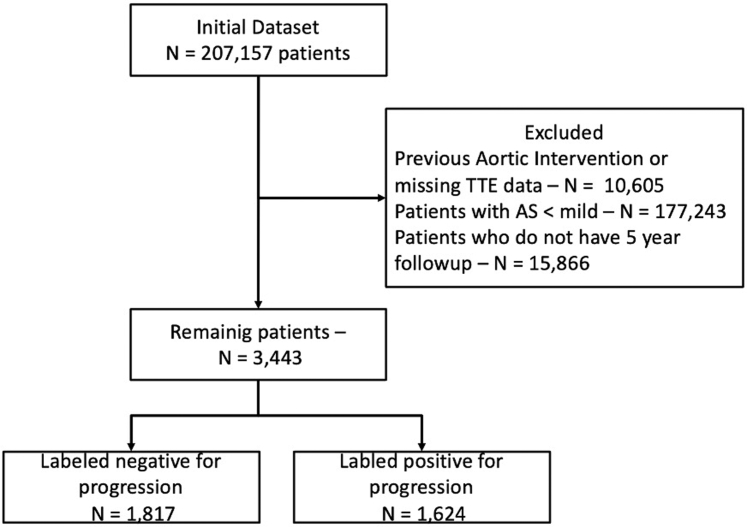
Patient Consort Flow diagram outlining patient inclusion, exclusion, and final study cohort.

This is a retrospective cohort study of all adult patients (>18 years old) who completed an echocardiographic evaluation at the Rabin Medical Center, located in Petah Tikva, Israel. Rabin Medical Center is a large cardiology referral center in Israel, with ∼30,000 echocardiographic tests performed each year. The echocardiographic reports were the source for this study. The severity of AS was assessed through an integrated approach combining hemodynamic, morphological, and semi-quantitative parameters, in line with the guidelines of the American Heart Association and the European Society of Cardiology. Quantitative classification of severe AS was based on a combination of valve hemodynamics (Vmax >4.0 m/s, mean transvalvular gradient ≥40 mm Hg), calculated AVA (<1.0 cm^2^ or AVA indexed to body surface area <0.6 cm^2^/m^2^), and a dimensionless velocity index <0.25, with additional consideration of valve morphology on echocardiography. In discordance between AVA and flow-gradient parameters, the final classification was determined by expert consensus within the echocardiography team. The degree of aortic valve stenosis (ASgrade) was categorized by the readers as follows: none (0), trivial (1), mild (2), mild-to-moderate (3), moderate (4), moderate-to-severe (5), and severe (6). Exams with no/trivial AS or severe AS on the first exam and patients who underwent aortic valvular interventions were excluded from the analysis (Figure 1). Additional baseline demographic and clinical data were retrieved from the computerized records. Diagnoses were based on computerized hospitalization records (International Classification of Diseases-9th Revision codes) (Supplemental Table 1). From an initial database of 529,751 echo exams, we first eliminated patients with no explicit mention of aortic valve status in the echo report or patients after a previous aortic valvular intervention. After that, we removed patients with no or trivial AS and selected only patients with a minimum of 5 years of follow-up. Based on the echo report, we identified 9,330 exams from 3,443 unique patients and labeled patients as positive or negative for progression to severe AS. Clinical data was available for 99.9% (n = 3,441) of the cohort. The institutional review board of the Rabin Medical Centre approved this study based on strictly maintaining participants' anonymity during database analyses. No individual consent was required or obtained.

### Model development

The machine learning model was developed using the CatBoost algorithm, which is well-suited for handling categorical data and has been shown to perform effectively in various medical datasets. The target variable for our model was the binary label indicating whether a patient's AS progressed to the severe stage (ASgrade = 6) within 5 years (labeled as 1) or not (labeled as 0). We chose to avoid binning follow-up intervals or applying a discrete-time survival model to ensure consistent label definitions and reduce the risk of introducing bias from irregular sampling.

The dataset used for model development comprised echocardiographic data from 9,330 visits after rigorous preprocessing. The target variable (Label) was separated from the features, which included a range of parameters available from the reports, such as: age, gender, heart rate, systolic blood pressure, diastolic blood pressure, height, weight, Aortic Annulus (AoAnulus), Ascending Aorta (AscAort), Aortic Root (AoRoot), Left Atrial Diameter (LaDiam), Left Atrial Area (LaArea), Interventricular Septum (IVS), Left Ventricular End-Diastolic Diameter (LVEDD), Left Ventricular Posterior Wall (LVPW), Left Ventricular End-Systolic Diameter (LVESD), Left Ventricular Function (Lvfunction), Ejection Fraction (EF), Estimated Ejection Fraction (EstimatedEF), Aortic Regurgitation Grade (ARgrade), AVA, Aortic Stenosis Maximum Gradient (ASmax), Aortic Stenosis Mean Gradient (ASmean), Doppler Velocity Index, Acceleration Time to Ejection Time ratio (AT_ET), Energy Loss (Energy_loss), Left Ventricular Outflow Tract Diameter (LVOTD), and more.

To prevent data leakage and ensure the model's performance would be evaluated on completely unseen patients, we grouped the data using an anonymized unique patient identifier and employed a GroupShuffleSplit strategy. This approach ensures that data from the same patient does not appear in training and test sets. The data was split into training and test sets using a 90:10 ratio, resulting in distinct cohorts for model training and validation (8,356 visits in the training set, 974 in the test set).

The CatBoost classifier was trained on the training set with the following hyperparameters: 1,000 iterations, a depth of 10, a learning rate of 0.01, and a loss function of Logloss. Class imbalance in the dataset was addressed by applying auto_class_weights = “Balanced” to ensure the model correctly learned from both classes (progression and nonprogression to severe AS).

The trained model was then evaluated on the test set. Predictions were generated for both the binary outcomes and the probabilities of progression. The model's performance was assessed using a variety of metrics, including accuracy, positive predictive value (PPV), sensitivity, F1-score, specificity, and the area under the receiver operating characteristic curve (AUC-ROC). A calibration curve was generated to assess the reliability of the predicted probabilities. Bootstrapping with 1,000 iterations was employed to calculate confidence intervals for all performance metrics, providing a robust estimate of the model's uncertainty and reliability. The calculated confidence intervals showed that the model's predictions were stable and consistent across different samples. Missing values were handled natively by the CatBoost algorithm, eliminating the need for imputation.

A standard probability threshold of 0.5 was used to dichotomize model outputs. SHAP (SHapley Additive exPlanations) values were computed using a TreeExplainer to gain insights into the model's decision-making process. A beeswarm plot was generated to visualize the most important features driving the model's predictions, providing interpretable insights into which echocardiographic parameters were most influential in predicting AS progression. In addition, to ensure the model's generalizability, we performed 10-fold cross-validation using a GroupKFold approach. The data was again split based on patient IDs to prevent data leakage.

To further asses the models' performance, we conducted another analysis using standard imputation methods (mean for continuous and mode for categorical variables), applied separately to the training and test sets to prevent data leakage. We then trained both a CatBoost model and a logistic regression model on the same imputed dataset and compared their performance using bootstrapped metrics with 95% CIs.

### Error analysis

We conducted an extended error analysis to examine potential patterns among the confusion matrix groups (true positives, false positives, true negatives, and false negatives). Specifically, we systematically analyzed each feature in the test dataset to assess its relationship with misclassification. For categorical variables, we computed chi-squared contingency tests and Cramér's V to assess the strength and significance of associations with confusion matrix groupings. We applied the Kruskal–Wallis test for continuous variables to identify statistically significant differences among groups. Where significance was detected (*P* < 0.05), we performed post hoc pairwise comparisons using Dunn's test, and for significant pairs, we calculated bootstrap-based effect sizes with 95% confidence intervals. We also generated bar plots (for categorical features) and box plots (for continuous features) to visualize these relationships. All figures are available in the Supplemental File.

### Statistical analysis

Continuous variables were expressed as mean ± SD if normally distributed or median (IQR) if skewed. Categorical variables were presented as frequency (%). Continuous data were compared with the Student's *t*-test, and categorical data were compared using the chi-square or Fisher exact tests. All analyses were performed in R software version 4.4.0 (R Foundation for Statistical Computing) and Python version 3.10. An association was considered statistically significant for a 2-sided *P* value of < 0.05.

The dataset included a wide range of clinical and echocardiographic parameters. To refine this dataset, we first applied a series of filters to exclude patients with prosthetic aortic valves, aortic valve-in-valve procedures, repaired mitral valves, mitral valve-in-valve procedures, prior transcatheter aortic valve implantation, and intraoperative echocardiograms. In addition, only transthoracic echocardiograms with a documented aortic stenosis grade (ASgrade) were retained, reducing the dataset to 444,917 visits from 196,552 patients.

Next, we focused on identifying visits in which patients had an aortic stenosis grade (ASgrade) of 2 or higher. We aimed to label each visit based on whether the patient progressed to severe aortic stenosis (ASgrade = 6) within 5 years. Visits were retained if there was a documented progression to severe AS within this period or documented evidence in the dataset that there was no progression after at least 5 years. Visits in which neither condition was met were excluded from further analysis. After the labeling process, we excluded the initial visits with ASgrade = 6. This labeling process resulted in a final dataset of 9,330 visits from 3,443 patients, with 56.12% labeled positive for progression to severe AS over time (Figure 1).

## Results

### Patient characteristics

The initial comprehensive dataset comprised 529,751 echocardiographic visits from 207,157 patients. We performed a final analysis on a dataset of 9,330 visits from 3,443 patients. The cohort's median age was 74 (interquartile range 66-80), and 49% were males. Sixty percent of the patients suffered from hypertension, chronic kidney disease was present in 14.6%, and congestive heart failure in 23%. A history of myocardial infarction was noted in 44.8% of the cohort, and peripheral vascular disease in 9.6%. Further baseline patient characteristics are presented in Table 1. The baseline echocardiographic parameters demonstrated a mean AVA of 1.06 ± 0.54 cm^2^, a mean gradient of 22.2 ± 11.7 mm Hg, and a peak aortic pressure of 38.4 ± 17.0 m/s. Most patients had preserved LVEF (median 46.5% ± 25.0%).

**Table 1:** Baseline Characteristics of the Study Population

	Overall (N = 3,441)	Did Not Progress to Severe as (n = 1,817)	Progressed to Severe as (n = 1,624)	*P* Value
Male	1,694 (49.2)	882 (48.6)	812 (50.0)	0.421
Age, y	74.2 (66.2-80.2)	71.8 (63.5-77.5)	76.8 (69.5-82.7)	<0.001
Body mass index, kg/m^2^	28.0 (25.2-31.6)	28.1 (25.3-32.0)	27.9 (24.8-31.0)	0.327
Creatinine	0.9 (0.8-1.1)	0.9 (0.7-1.1)	0.9 (0.8-1.3)	<0.001
Death	2,315 (67.3)	1,096 (60.3)	1,219 (75.1)	<0.001
Hypertension	2,051 (59.6)	997 (54.9)	1,054 (64.9)	<0.001
Chronic kidney disease	502 (14.6)	169 (9.3)	333 (20.5)	<0.001
Congestive heart failure	664 (23.0)	295 (19.0)	369 (27.7)	<0.001
Cerebrovascular accident	794 (27.4)	349 (19.2)	445 (27.3)	<0.001
Myocardial infarction	1,367 (44.8)	666 (42.5)	701 (47.5)	0.005
Diabetes mellitus	1,147 (39.3)	539 (35.2)	608 (44.5)	<0.001
Dyslipidemia	2,015 (58.6)	992 (54.6)	1,023 (63.0)	<0.001
Chronic obstructive pulmonary disease	601 (21.4)	344 (19.0)	257 (23.7)	0.006
Peripheral vascular disease	329 (9.6)	134 (7.4)	195 (12.0)	<0.001
Atrial fibrillation	716 (20.8)	344 (18.9)	372 (22.9)	0.012
Smoker (current or former)	810 (23.5)	409 (22.5)	401 (24.7)	0.168

Values are n (%) or median (IQR).

The mean time to deterioration from mild/moderate to severe AS was 2.62 ± 3.87 years, and patients underwent an average of 2.80 echocardiographic exams between the first exam and the diagnosis of severe AS. In our model, some patient variables showed small but statistically significant differences between the test and the training sets. (eg, age: 71.9 vs 70.5 years, *P* = 0.0032). These differences were mostely small in magnitude and likely not clinically meaningful. For instance, a difference of ∼0.03 m^2^ in body surface area is unlikely to introduce meaningful bias. Further comparison of variables between the train and test splits can be seen in Table 2.

**Table 2:** Echo Report Characteristic Differences Between the Test and Train Split

	Train	Test	*P* Value
Negative label	3,655 (43.7%)	439 (45.1%)	0.4328
Age	71.92	70.5	0.0032
Male	51.50%	53.40%	0.2638
Body surface area	1.81	1.78	0.0095
Aortic root anulus	2.39	2.33	0.631
Ascending aort	3.53	3.54	0.7609
Left atrial diameter	4.33	4.41	0.4071
Left atrial area	23.82	25.39	<0.001
Intraventricular septum	1.19	1.17	0.1806
Left ventricular end diastolic diameter	4.69	4.66	0.273
Left ventricular end systolic diameter	2.93	2.95	0.5789
Ejection fraction	46.87	46.86	0.998
TAPSE	20.14	19.25	0.0966
Aortic valve area	1.05	1.07	0.22
Maximal aortic valve gradient	38.36	38.37	0.9797
Mean aortic valve gradient	22.21	22.25	0.916
Stroke volume	76.41	74.83	0.3407
Dimensionless velocity index	0.3	0.3	0.2788
Energy_loss	1.19	1.24	0.3199
Maximal mitral valve gradient	13.21	13.66	0.2921
Mean mitral valve gradient	5.68	5.54	0.5736
Maximal tricuspit valve gradient	46.33	45.15	0.0358
Inferir vena cava diameter	2.28	2.37	0.0411

AVA = aortic valve area; TAPSE = tricuspid annular plane systolic excursion.

### Model performance

**Figure 2:**
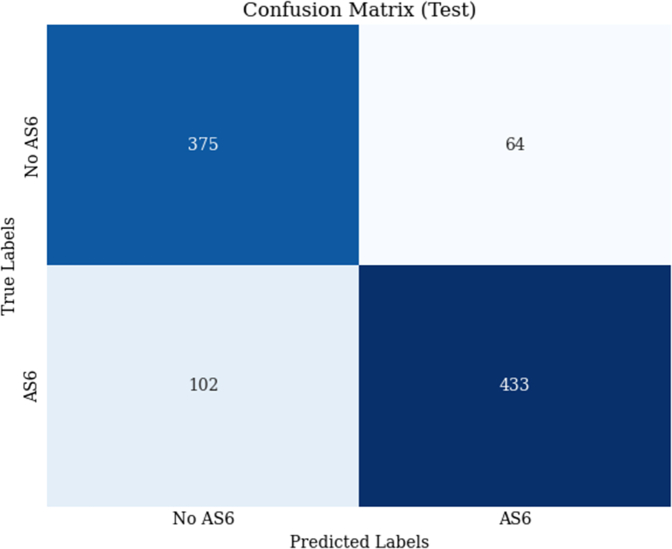
Confusion Matrix Model classification results showing true vs predicted outcomes.

The model's performance was assessed using a confusion matrix, a calibration curve, a ROC curve, and various cross-validation metrics. The confusion matrix (Figure 2) summarizes the model's classification performance. The model correctly identified 375 out of 439 cases that did not deteriorate to severe AS during the follow-up period—“No AS6” cases (true negatives) and misclassified 64 cases as “AS6” (false positives). For the cases that did deteriorate to severe AS during the follow-up period – “AS6” class, the model correctly identified 433 out of 535 cases (true positives) and misclassified 102 cases as “No AS6” (false negatives), indicating balanced performance across both classes.

#### Calibration curve and Integrated Calibration Index

**Figure 3:**
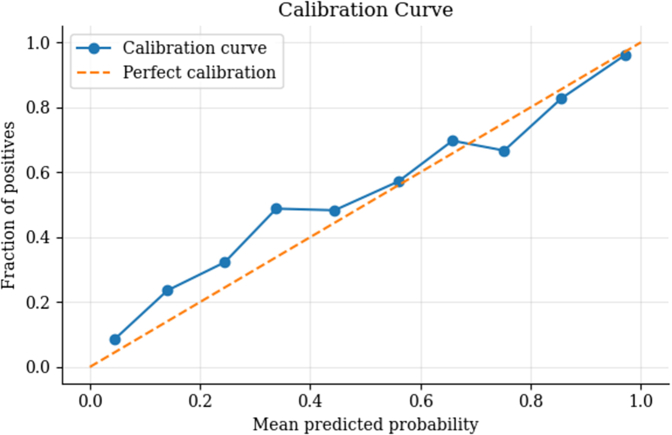
Calibration Curve Agreement between predicted probabilities and observed outcomes.

The calibration curve (Figure 3) indicates that the model's predicted probabilities closely align with the observed outcomes across various thresholds. The Integrated Calibration Index of 0.0576 demonstrates good overall calibration, reflecting minimal deviation from perfect calibration.

#### Receiver operating curve

**Figure 4:**
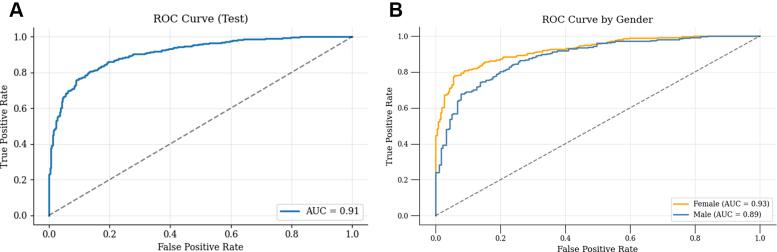
Receiver Operator Curve (A) Receiver operator curve (ROC) displaying model discrimination performance. (B) Receiver operator curve by sex—ROC curve displaying model discrimination performance by sex.

The ROC curve (Figure 4A) illustrates the model's discrimination ability, with an AUC of 0.91 (95% CI: 0.89-0.91), indicating excellent performance in distinguishing between patients who did and did not deteriorate to severe AS.

#### Overall model performance metrics

**Figure 5:**
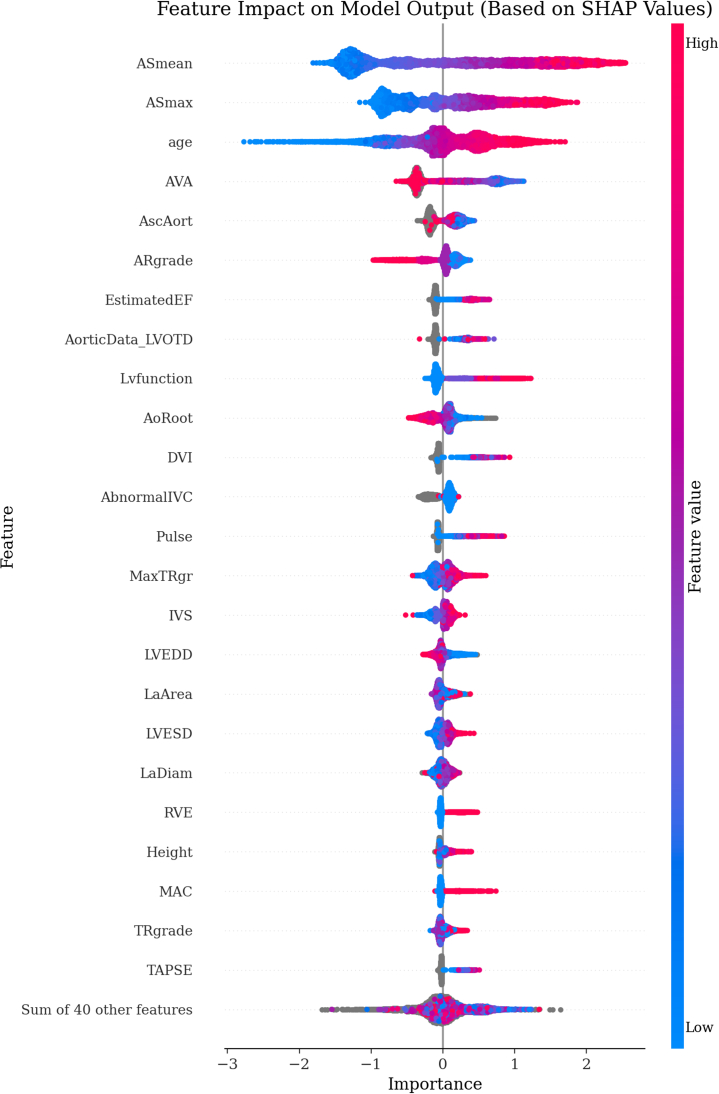
SHAP Analysis SHAP summary plot highlighting key predictors of model output. SHAP = SHapley Additive exPlanations.

**Figure 6:**
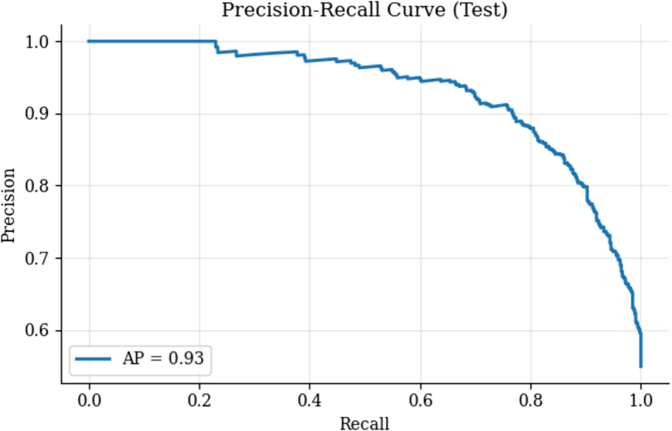
Precision-Recall Curve Trade-off between precision and recall across probability thresholds.

The model's performance metrics are as follows: model accuracy 0.83 (95% CI: 0.81-0.85), model PPV 0.87 (95% CI: 0.84-0.90), model sensitivity: 0.81 (95% CI: 0.78-0.84). Model F1 score: 0.84 (95% CI: 0.81-0.86). Model AUC 0.91 (95% CI: 0.89-0.93) Model specificity: 0.85 (95% CI: 0.82-0.89). We performed a cross-validation to confirm the model's robustness, with average metrics closely aligning with the main model evaluation results: average model accuracy 0.86. average model PPV 0.89. Average model sensitivity is 0.86, average Model F1-score 0.87, and average model AUC 0.93, average model specificity is 0.86. The SHAP beeswarm plot (Figure 5) illustrates the impact of each feature on the model's output. Each point represents a value for a single observation. The position of the points along the x-axis reflects the SHAP value, indicating the magnitude and direction of the impact on the model's prediction. Features are listed in descending order of importance based on their average absolute SHAP values. Table 3 lists the features in their order of importance. A precision-recall curve (Figure 6) shows that the area under the precision-recall curve (average precision, AP) was 0.93, indicating excellent discriminative ability in identifying positive cases.

**Table 3:** SHAP Analysis

Echocardiographic Parameter	SHAP Value
ASmean	1.077822289794323
ASmax	0.62375383
Age	0.4913985235768867
AVA	0.44320269701948306
AscAort	0.17975358457736362
ARgrade	0.1712929659241511
EstimatedEF	0.1708235979052965
LVOTD	0.16848189155514703
Lvfunction	0.15396063698196588
AoRoot	0.1337162296288667

The most influential features for the model's predictions include the mean AS gradient, maximum AS gradient, age, AVA, ascending aorta diameter, and aortic regurgitation grade. High values of maximum AS gradient (indicated by red points) are associated with higher model outputs, suggesting that increased AS gradients significantly contribute to the predicted outcome. Conversely, lower values of features such as AVA and ascending aorta diameter (indicated by blue points) are associated with lower model outputs.

**Figure 7:**
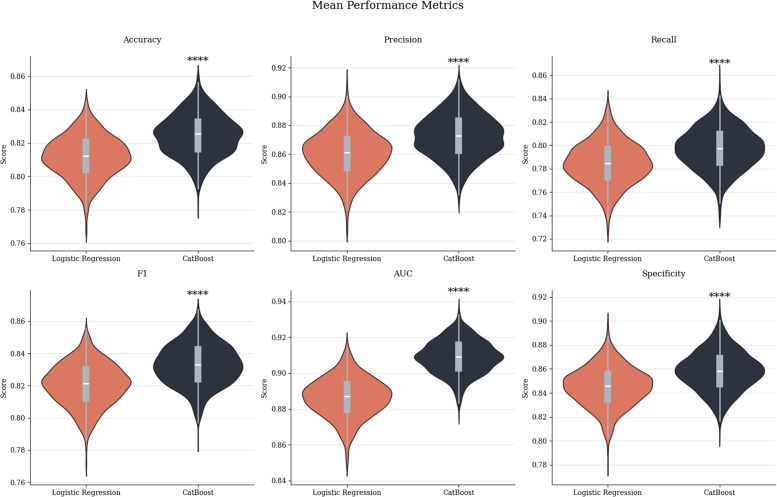
Comparison of Logistic Regression and Catboost Models Performance comparison between logistic regression and CatBoost models, highlighting differences in predictive accuracy.

When comparing the model performance to a logistic regression model trained using standard imputation methods, CatBoost significantly outperformed logistic regression across all metrics. The difference in AUC was particularly notable: CatBoost AUC = 0.910 (95% CI: 0.890-0.926) vs logistic regression AUC = 0.886 (95% CI: 0.864-0.907), with a large effect size (Cohen's d = 1.6, *P* < 0.001). Other metrics, including accuracy, precision, recall, F1-score, and specificity, were also significantly higher for CatBoost (*P* < 0.001 for all) (Figure 7). Further details of this analysis are available in the Supplemental Appendix.

### Error analysis

To assess potential gender-based performance differences, we performed a stratified analysis by evaluating the model separately on the male and female subgroups in the test set. We computed classification metrics for each subgroup—including accuracy, precision, recall, F1-score, AUC, and specificity—along with 95% confidence intervals using bootstrapping. ROC and precision-recall curves were also generated to visualize performance in each group. Our analysis revealed that the model performed well in both subgroups but showed slightly better performance in females. For the female subgroup, the model achieved an accuracy of 0.86 (95% CI: 0.83-0.88), AUC of 0.93 (95% CI: 0.90-0.95) (Figure 4B), and specificity of 0.90 (95% CI: 0.86-0.94). In comparison, the male subgroup showed an accuracy of 0.80 (95% CI: 0.76-0.83), AUC of 0.89 (95% CI: 0.85-0.91) (Figure 4B), and specificity of 0.78 (95% CI: 0.72-0.84). The recall was consistent across sexes (0.81), but the higher specificity and AUC in females suggest slightly improved discrimination and reduced false-positive rates in this group.

## Discussion

Current guidelines^2^ do not offer distinct patient-centered follow-up recommendations for patients with mild or moderate AS, providing only a general recommendation that patients with moderate degenerative AS should be re-evaluated at least annually, and younger patients with mild AS and no significant calcification may be followed up every 2–3 years.^12^ This approach may cause uncertainty for the treating practitioners, result in unnecessary exams, and miss rapidly progressing cases. It may also lead to gender disparities, as women with AS often have higher mortality rates due to late diagnosis and less-frequent referrals for intervention.^13^ While several recent studies have applied AI to detect existing severe AS, the novelty of our approach lies in its predictive focus, identifying patients at risk of progression from mild/moderate to severe AS rather than classifying current severity. This forward-looking application allows clinicians to proactively tailor surveillance intensity and potentially intervene earlier, based on individualized risk rather than static severity thresholds.

Our study presents a new AI-based model for predicting the progression of AS from mild/moderate to severe within 5 years or less using solely echocardiographic data. We used data readily available from the echocardiography report, and the model requires no additional clinical information. Our model showed strong performance, with an AUC of 0.91, a PPV of 0.87, and an Integrated Calibration Index of 0.0576, indicating high accuracy and good calibration. These results suggest that the AI model could be a reliable tool in clinical practice for early identification of patients at risk of rapid AS progression, potentially allowing for more timely interventions and better follow-up schemes. In addition, when comparing the model performance to a logistic regression model, our model significantly outperformed logistic regression across all metrics.

Our research findings align with recent studies highlighting the potential of AI in advancing the diagnosis and management of AS—several papers focused on severe AS-detection AI tools applied to echocardiography. A study by Holste et al^14^ used deep learning to develop and externally validate a model for severe AS detection based on single-view 2D echocardiography; their model achieved an AUC-ROC over 0.95 in both geographically and temporally distinct test sets. Another study by Wessler^8^ focused on developing a model for the automated detection of AS from an imaging dataset. This study showed that trained neural networks can detect any AS from 2D images with an AUC of 0.96. They could also differentiate between nonsignificant (no, mild, mild-moderate) AS from significant (moderate or severe) AS with an AUC of 0.86. Another study by Cohen-Shelly et al^15^ showed the potential for using AI models to detect severe AS from nonecho media, in this case, ECG exams, developing a model with an AUC of 0.85 and a sensitivity, specificity, and accuracy of 78%, 74%, and 74%, respectively. The model's performance was improved when sex and age data were added. In addition, notably, patients who were concluded as false-positive had twice the risk of developing moderate or severe AS in 15 years compared with true negative. In a study by Sanabria et al,^16^ an attempt was made to develop a prediction model of AS progression based on 303 patients enrolled in the PROGRESSA trial. Their study achieved an AUC of only 0.83 for 5-year progression prediction, possibly due to the study's small size.

Playford et al^17^ also examined AI-based echocardiographic assessments and found that AI could accurately identify patients with severe AS, even without relying on left ventricular outflow tract measurements, prone to measurement errors and variability. Our model further confirms this work by highlighting key parameters such as AS gradients (mean and max), age, and aortic regurgitation grade, emphasizing the crucial role of AI in improving the diagnostic process by reducing the dependence on potentially flawed measurements. The novelty of our model is its use of echocardiographic data only, thus not requiring electronic-medical-record integration for utilization. It also opens the door for customized follow-up programs based on specific patient risk stratification.

### Clinical implications and potential applications

Our study adds to the existing work by demonstrating that an AI-based model can do more than diagnose severe AS. The novelty of our model lies in its ability to predict the progression from mild/moderate to severe AS. A good knowledge of patients likely to deteriorate to severe AS can reduce the number of patients missed by commonly used follow-up periods. Late diagnosis of symptomatic severe AS, especially when accompanied by heart failure,^18^ is detrimental to patients. Timely diagnosis of severe AS, even when asymptomatic, is also important as research shows that this state is not innocent. Patients suffer from increased mortality risk and require higher health care expenditure of 3.4 billion US dollars per annum than patients with no valvular disease.^19^ On the other hand, knowing which patients are less likely to deteriorate to severe AS can reduce the need for resource-intensive serial echocardiographic evaluations.

The potential clinical benefits of using AI in AS management are substantial. This predictive ability enables more precise, patient-tailored follow-up strategies. For example, instead of applying uniform follow-up intervals, an outpatient clinic could schedule high-risk patients identified by the model for echocardiographic reassessment every 6 to 12 months. In contrast, low-risk patients might safely extend their follow-up to 3 years, optimizing resource use without compromising care. Another advantage of this model from the broader healthcare perspective is the similar performance across genders (AUC 0.93 in females, 0.89 in males) (Figure 4B). The recall was consistent across sexes (0.81), but the higher specificity and AUC in females suggest slightly improved discrimination and reduced false-positive rates in this group.

From a systems perspective, the model is designed to integrate seamlessly into current workflows, requiring only echocardiographic data available in standard reports. This makes it compatible with existing echocardiography reporting software and adaptable to various healthcare systems without complex electronic medical record integration. Such implementation could enhance clinician decision-making at the point of care, streamline scheduling, and improve triage for cardiology referrals.

### Limitations and future directions

While our findings are promising, it is essential to note the limitations of our study. First, the retrospective nature of our data, obtained from a single-center cohort, may restrict the generalizability of our results. While we utilized rigorous methods to ensure the model's reliability, it is essential to conduct external validation using multicenter datasets to validate the model's applicability across diverse patient populations and clinical settings.

In addition, our model depends on high-quality, standardized echocardiographic data. Variability in echocardiographic techniques, equipment, and expertise across different institutions could affect the model's performance.

Furthermore, by focusing on echocardiographic data, we were able to build a model that can be quickly and easily integrated into current report-generation systems. However, this focus meant it did not include other potentially valuable data, such as biomarkers, cardiac magnetic resonance imaging, clinical history, or chronic medications, which could improve or alter its predictive accuracy. We are conducting further studies to explore the integration of multimodal data for a more comprehensive risk-stratification tool.

Our model was designed to provide predictions at the visit level, corresponding to the clinical scenario in which each echocardiographic examination represents a distinct opportunity for risk assessment. To rigorously prevent data leakage, patient-level grouping was applied when splitting the dataset for both the held-out test set and cross-validation folds. However, within each data partition, multiple visits from the same patient were treated as independent observations.

This approach reflects a deliberate trade-off between modeling complexity and alignment with the intended clinical prediction target: By treating visits as independent, the model remains focused on delivering actionable risk estimates for individual examinations, rather than modeling patient trajectories or aggregated outcomes across multiple visits. While this decision simplifies the model and supports its per-visit applicability, it does not account for potential intrapatient correlations among repeated visits. This recognized limitation could, in theory, be addressed through more complex hierarchical or longitudinal modeling approaches in future studies.

Finally, the ethical considerations of implementing AI in clinical practice must not be overlooked. It is crucial that the use of AI in guiding clinical decisions is transparent and that healthcare professionals are equipped to comprehend the rationale behind AI-generated predictions to ensure these tools' proper and effective use. In this paper, we attempted to address concerns about the “black-box” nature of AI models by adding the SHAP analysis to help interpret the model's predictions and by providing a complete description of the methodology and results of the model development (Central Illustration).

## Conclusions

Our team has developed an innovative model that can accurately predict the progression of AS from nonsevere to severe using data exclusively from echocardiography reports. This model offers a valuable tool for clinicians to identify high-risk patients and enhance management strategies. The model's impressive performance metrics suggest it could complement traditional diagnostic methods, potentially enabling earlier, more targeted diagnosis of severe AS, allowing early interventions that could lead to reduced morbidity and mortality. Moving forward, further research should aim to verify these findings in broader populations, integrate additional data sources, and evaluate the impact of AI-driven decision-making on patient outcomes.

Perspectives **COMPETENCY IN MEDICAL KNOWLEDGE:** Aortic stenosis is a progressive condition impacting a growing segment of older adults. Current follow-up guidelines are broad and may overlook patients with rapid deterioration. This study presents an artificial intelligence-driven model that forecasts the transition from mild or moderate aortic stenosis to severe disease utilizing solely echocardiographic data, facilitating earlier and more tailored clinical decision-making.

**TRANSITIONAL OUTLOOK:** The model showed outstanding performance (area under the curve 0.93) and calibration, and it relies solely on standard echocardiographic parameters, allowing easy integration into current reporting workflows. Utilizing SHapley Additive exPlanations values enhances explainability, tackling the black-box issues associated with artificial intelligence models. Future plans involve external validation in varied populations and integrating these models into clinical routines to strengthen patient triage, minimize diagnostic disparities, and optimize healthcare resource utilization.

## Funding support and author disclosures

The authors have reported that they have no relationships relevant to the contents of this paper to disclose.
