# Supplementary material for: Prediction of Aortic Stenosis Progression Using Artificial Intelligence: A Machine Learning Model
Source: JACC Adv. 2025 Aug 29;4(10):102121. doi: 10.1016/j.jacadv.2025.102121 (PMC12791866; doi:10.1016/j.jacadv.2025.102121)
Supplement: Supplementary data [file mmc1.docx]

Supplementary Appendix


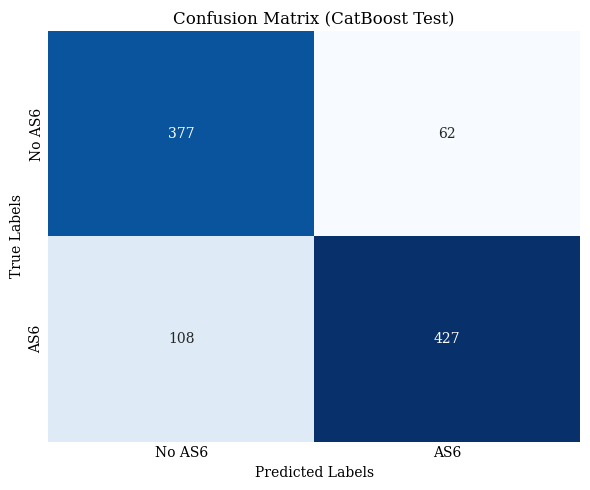


Supplementary Figure 1 - Confusion Matrix, Catboost Model


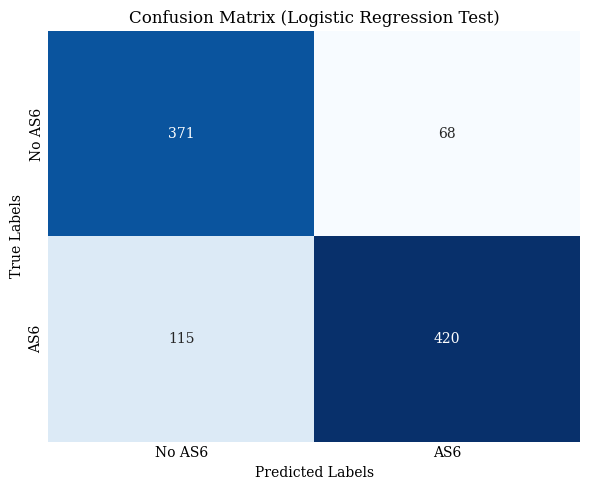


Supplementary Figure 2 - Confusion Matrix - LR model


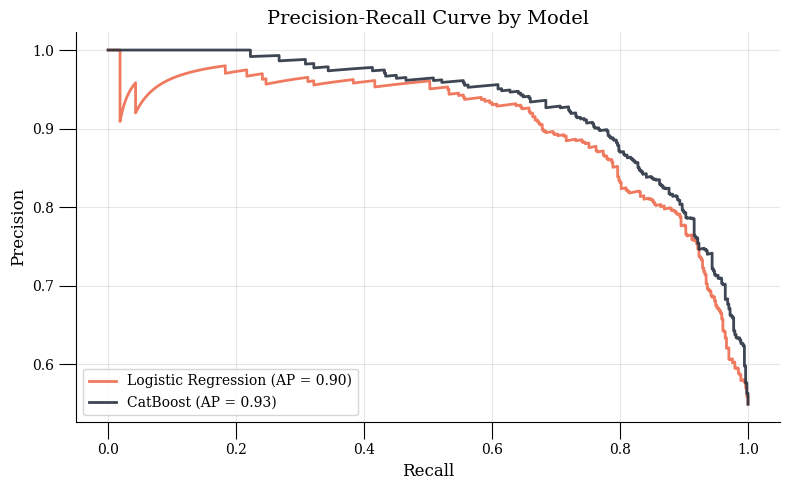


Supplementary Figure 3 - PR Curve - LR Vs. Catboost


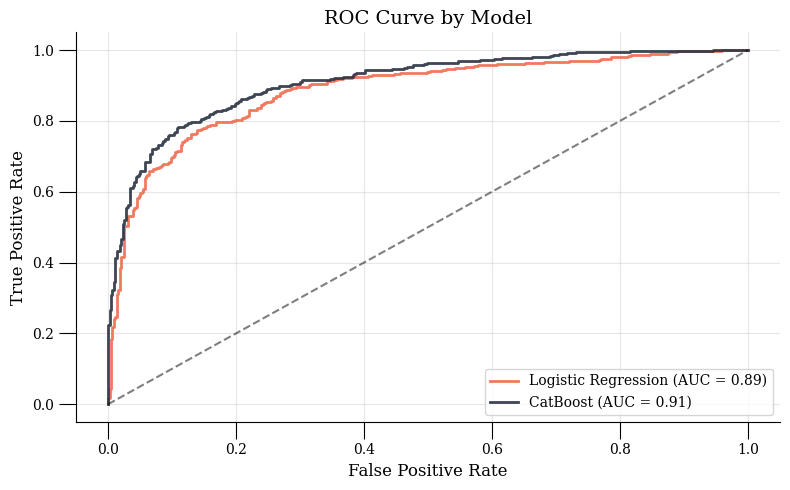


Supplementary Figure 45 - ROC Curve - LR Vs Catboost

| Condition | ICD-9-CM Codes |
| --- | --- |
| Hypertension | 401.9 |
| Chronic Kidney Disease (CKD) | 585.x |
| Congestive Heart Failure (CHF) | 428.0 |
| Cerebrovascular Accident (CVA) | 434.91 |
| Myocardial Infarction (MI) | 410.xx |
| Diabetes Mellitus (DM) | 250.xx |
| Dyslipidemia | 272.4,272.9 |
| Chronic Obstructive Pulmonary Disease (COPD) | 496 |
| Peripheral Vascular Disease (PVD) | 443.9 |
| Atrial Fibrillation (AF) | 427.31 |

Supplementary Table 1 - ICD Codes of diagnosis

| Metric | LR Mean (95% CI) | CB Mean (95% CI) | Cohen's d | t-test p | Wilcoxon p |
| --- | --- | --- | --- | --- | --- |
| accuracy | 0.812 (0.787–0.835) | 0.825 (0.800–0.848) | 0.726 | 4.39E-94 | 2.82E-81 |
| precision | 0.861 (0.829–0.889) | 0.873 (0.843–0.901) | 0.545 | 1.68E-58 | 1.22E-52 |
| recall | 0.785 (0.749–0.820) | 0.798 (0.763–0.830) | 0.516 | 2.54E-53 | 5.10E-49 |
| f1 | 0.821 (0.794–0.844) | 0.834 (0.807–0.858) | 0.675 | 1.55E-83 | 3.38E-73 |
| auc | 0.886 (0.864–0.907) | 0.910 (0.890–0.926) | 1.6 | 7.61E-278 | 1.24E-157 |
| specificity | 0.845 (0.810–0.876) | 0.859 (0.826–0.891) | 0.548 | 5.59E-59 | 9.18E-54 |

Supplementary Table 2 – Performance metrics of logistic regression model VS catboost model

| Feature | Train Missing (n) | Train Missing (%) | Test Missing (n) | Test Missing (%) |
| --- | --- | --- | --- | --- |
| Pulse | 6700 | 80.18 | 771 | 79.16 |
| SystBP | 8326 | 99.64 | 971 | 99.69 |
| DiastBP | 8326 | 99.64 | 971 | 99.69 |
| Height | 4498 | 53.83 | 508 | 52.16 |
| Weight | 4579 | 54.8 | 513 | 52.67 |
| BSA | 4571 | 54.7 | 518 | 53.18 |
| AoAnulus | 8311 | 99.46 | 966 | 99.18 |
| AscAort | 4070 | 48.71 | 457 | 46.92 |
| EF | 8296 | 99.28 | 960 | 98.56 |
| EstimatedEF | 6335 | 75.81 | 736 | 75.56 |
| diastolScore | 4909 | 58.75 | 595 | 61.09 |
| TAPSE | 7668 | 91.77 | 890 | 91.38 |
| AVA | 4497 | 53.82 | 503 | 51.64 |
| SV | 7089 | 84.84 | 818 | 83.98 |
| SVI | 7153 | 85.6 | 826 | 84.8 |
| AVAI | 7169 | 85.79 | 825 | 84.7 |
| DVI | 7107 | 85.05 | 824 | 84.6 |
| AT_ET | 8345 | 99.87 | 974 | 100 |
| Energy_loss | 7981 | 95.51 | 923 | 94.76 |
| Energy_loss_Index | 7993 | 95.66 | 925 | 94.97 |
| AorticData_LVOTD | 6020 | 72.04 | 688 | 70.64 |
| MSmax | 6520 | 78.03 | 725 | 74.44 |
| MSmean | 6537 | 78.23 | 726 | 74.54 |
| AbnormalIVC | 2735 | 32.73 | 312 | 32.03 |
| IVCdiam | 7549 | 90.34 | 854 | 87.68 |

Supplementary Table 3 - Features with more than 30% missing values
